# A phase II study of oral piritrexim in recurrent high-grade (III, IV) glioma.

**DOI:** 10.1038/bjc.1995.407

**Published:** 1995-09

**Authors:** N. M. Bleehen, H. V. Newman, R. P. Rampling, J. R. Ramsay, J. T. Roberts, P. Bedford, A. B. Nethersell

**Affiliations:** University Department, Addenbrooke's Hospital, Cambridge, UK.

## Abstract

Piritrexim is a lipid-soluble drug which is as effective an inhibitor of dihydrofolate reductase as methotrexate. Phase I and II studies have indicated activity in some tumour types. Because of its lipophilicity we have conducted a phase II study in recurrent high-grade malignant glioma (grades III and IV). Twenty-seven patients were treated with 25 mg p.o. three times daily for five consecutive days, repeated weekly, with provision for dose escalation or reduction according to toxicity. Five patients received less than 4 weeks' treatment because of disease progression or death. Twenty-two patients were evaluable for response. One complete and one partial response was seen (duration 262+ and 241+ weeks) and 13 patients had static disease for a median duration of 13 weeks (range 7-35). The major toxicity was myelosuppression. This response rate of 9% of evaluable patients is much lower than that seen for some conventionally used drugs and we conclude that piritrexim is unlikely to be of value in the management of high-grade gliomas.


British Journal of Cancer (1995) 72, 766-768

© 1995 Stockton Press All rights reserved 0007-0920/95 $12.00

SHORT COMMUNICATION

### A phase II study of oral piritrexim in recurrent high-grade (III, IV) glioma

NM Bleehen[1], HVF Newman[2], RP Rampling[3], JR Ramsay[1,*], JT Roberts[4], P Bedford[5] and ABW Nethersell[5]

[1]*University Department and MRC Unit of Clinical Oncology and Radiotherapeutics, Addenbrooke's Hospital, Cambridge CB2 2QQ, UK;* [2]*Department of Radiotherapy and Oncology, Bristol Royal Infirmary, Bristol BS2 8HW, UK;* [3]*Beatson Oncology Centre, Western Infirmary, Glasgow G11 6NT, UK;* [4]*Northern Centre for Cancer Treatment, Newcastle General Hospital, Newcastle upon Tyne NE4 6BE, UK;* [5]*Department of Clinical Oncology, The Wellcome Foundation Ltd, Langley Court, South Eden Park Road, Beckenham, Kent BR3 3BS, UK.*

**Summary** Piritrexim is a lipid-soluble drug which is as effective an inhibitor of dihydrofolate reductase as methotrexate. Phase I and II studies have indicated activity in some tumour types. Because of its lipophilicity we have conducted a phase II study in recurrent high-grade malignant glioma (grades III and IV). Twenty-seven patients were treated with 25 mg p.o. three times daily for five consecutive days, repeated weekly, with provision for dose escalation or reduction according to toxicity. Five patients received less than 4 weeks' treatment because of disease progression or death. Twenty-two patients were evaluable for response. One complete and one partial response was seen (duration 262+ and 241+ weeks) and 13 patients had static disease for a median duration of 13 weeks (range 7-35). The major toxicity was myelosuppression. This response rate of 9% of evaluable patients is much lower than that seen for some conventionally used drugs and we conclude that piritrexim is unlikely to be of value in the management of high-grade gliomas.

**Keywords:** glioma; piritrexim; dihydrofolate reductase inhibitor

Piritrexim (2, 4-diamino-6-(2, 5-dimethoxybenzyl)-5-methylpyridol[2,3-*d*]pyrimidine; BW 301U; PTX) is a lipid-soluble inhibitor of dihydrofolate reductase (DHFR; Duch *et al.*, 1982; Sedgwick *et al.*, 1982; Sigel *et al.*, 1987). It enters cells rapidly, is not polyglutamated intracellularly and is as potent an inhibitor of DHFR *in vitro* as methotrexate (Duch *et al.*, 1982). Activity against human tumour cells has been demonstrated using the human tumour cloning assay in lung, ovary, colon and breast cancer (Neuenfeldt *et al.*, 1982; Marshall *et al.*, 1985).

Intravenous administration causes peripheral phlebitis and, as oral bioavailability is around 75%, oral dosing has been recommended (Weiss *et al.*, 1989). A phase I study using prolonged oral low-dose schedules has demonstrated an acceptable regimen of 25 mg three times daily for 5 days out of 7 for three consecutive weeks followed by 1 week without PTX (Feun *et al.*, 1991). Myelosuppression was the main dose-limiting toxicity. A paediatric phase I study has also been reported (Adamson *et al.*, 1990).

Several phase II studies have now been reported. Anti-tumour activity has been seen in melanoma (Feun *et al.*, 1991), head and neck cancer (Uen *et al.*, 1992), soft-tissue sarcoma (Schiesel *et al.*, 1992) and metastatic urothelial cancer (De Wit *et al.*, 1993*a*). Some activity was also seen in non-small-cell lung cancer (Kris *et al.*, 1987) and in breast cancer (de Vries *et al.*, 1993). However, little activity was seen in a further study in head and neck cancer using a very different schedule and in combination with methotrexate (Vokes *et al.*, 1991). Because of the lipophilicity of PTX, we have carried out a multicentre phase II study in recurrent adult high-grade (grade 3 and 4) gliomas to assess its potential efficacy.

Correspondence: NM Bleehen
*Present address: Queensland Radium Institute, Royal Brisbane Hospital, Herston, Australia Q4029.
Received 10 March 1995; revised 4 April 1995; accepted 7 April 1995

### Patients and methods

### *Patients*

Eligible patients were required to have histologically confirmed high-grade (Kernohan grade III or IV) malignant glioma which had relapsed after treatment by surgery, radiotherapy or chemotherapy or a combination of these modalities. Other entry criteria were (a) age 18-75 years; (b) WHO performance status $\leqslant 3$; (c) neurological status $\leqslant 3$ (MRC scale); (d) estimated life expectancy of at least 8 weeks; (e) at least 6 weeks elapsed since radiotherapy or previous chemotherapy; (f) no adjustment to steroid dose within the previous week; (g) leucocyte count $>4\times10^9$ $l^{-1}$ and platelet count $>100\times10^9$ $l^{-1}$; (h) serum creatinine level $<140$ mmol $l^{-1}$; (i) serum bilirubin $<25$ μmol $l^{-1}$; (j) aspartate transaminase (AST) or alanine transaminase (ALT) $<2$ times normal; (k) alkaline phosphatase (ALP) $<2$ times normal. The protocol was approved by the local research ethnics committee of the participating centres. Informed consent was obtained from all patients before entry in the study.

### Study design

Capsules of PTX were administered orally each day for five consecutive days and repeated weekly after 2 days' rest. An initial dose of 25 mg three times daily was continued through the first four weekly cycles provided no toxicity (other than grade 1 haemoglobin toxicity) was seen. In the absence of any toxicity the dose was escalated to 25 mg four times daily. This dose escalation schema, to titrate against toxicity, had been defined in the phase I studies and used in phase II studies (e.g. De Wit *et al.*, 1993*a*; de Vries *et al.*,1993). If grade 1 myelotoxicity was seen the dose remained unchanged. If grade 2 myelotoxicity was encountered within the first 3 weeks (or grade 3/4 at any time) dosing was delayed until recovery, at which point drug administration was resumed at 25 mg t.d.s. for 4 days each week. A further reduction to 25 mg twice daily for 4 days was used in subsequent cycles if the toxicity was repeated. Antiemetics at all doses of the chemotherapy were used to control nausea. Patients continued on PTX until disease progression or unacceptable toxicity.

Patients were seen weekly for assessment and for dose adjustment during the first 12 weeks of the study and 4 weekly thereafter. Physical and neurological assessment and of disease response was carried out 4 weekly. Computerised tomographic (CT) and/or magnetic resonance imaging (MRI) examination was carried out to provide supplementary evidence for objective response.

The Medical Research Council (MRC) neurological scale was defined as: grade 0, no neurological deficit; grade 1, some neurological deficit but function adequate for work; grade 2, neurological deficit causing moderate functional impairment; grade 3, neurological deficit causing major functional impairment such as inability to use limb(s), gross speech or visual disturbance; grade 4, no useful function with inability to make conscious responses. An objective response was defined as improvement of one or more neurological symptoms to improve the neurological status by one grade on the MRC scale. In addition, there should be no new neurological deficits and the dose of steroids should be constant. This objective response could be documented with or without a tumour response as seen by imaging. This imaging response was defined as a reduction of 50% or more in tumour size, as the product of the two largest perpendicular diameters of the lesion measured by CT or MRI. Stable disease was defined as no change in neurological status irrespective of change in tumour size but with constant steroid dosage. Progressive disease was recorded when there was a deterioration of neurological status and/or increase in steroid dose. To be evaluable for response, patients were required to have completed at least four cycles of treatment. The period of objective response was defined from the date first observed to the date of the first observation of progressive disease. Toxicity was recorded as defined by the WHO toxicity scale (WHO, 1979).

### Results

Twenty-eight patients were entered into the study, but one was ineligible by reason of histology (grade 1) and was withdrawn. The characteristics of the remaining 27 patients are shown in Table I. Five patients were not evaluable for response as they received less than 4 weeks' treatment, four because of rapid clinical deterioration and one died in status epilepticus after three weekly courses. These non-evaluable patients remain included in the assessment of toxicity. Twelve patients had previously received chemotherapy with either the PCV regimen (procarbazine, CCNU and vincristine), or single-agent nitrosoureas (CCNU/BCNU). Three patients relapsing after PTX received subsequent chemotherapy, two with PCV and one with CCNU alone.

A total of 226 weekly courses was given in the 27 patients. The median number of courses per patient was 7 (range 1–27) and the median duration of treatment was 8 weeks (range 1–37). The frequency of daily dosage per week was increased from the initial three times per day to four times per day in 17 patients and five times per day in eight patients.

There were two responders in the 22 evaluable patients (9%), or 7% of the 27 eligible patients entered into the study. One of the 22 evaluable patients had a good clinical and radiological complete response (CR) with a duration of response of 262+ weeks. A second patient had a very good partial response (PR) both radiologically and clinically, receiving 17 courses of PTX before it was stopped because of persistent mucositis. On subsequent relapse he responded (PR) to CCNU and remains stable 241+ weeks after first commencing PTX.

Of the remaining assessible patients, 13 (59% of evaluable, 48% of total eligible) were stable for a median time of 13 weeks (range 7–35). Disease progression following the start of PTX was seen in the remaining seven patients. The median survival time of all 27 eligible patients from the start of PTX treatment was 26 weeks (range 4–241+), and of the 22 assessible patients 30 weeks (range 4–241+).

Details of toxicity are given in Table II. The main toxicity was haemopoietic with grade 1 or 2 leucopenia in 8/27 (30%), and neutropenia in 5/27 (19%). Thrombocytopenia grade 1 or 2 occurred in 5/27 (19%), grade 3 in one patient and grade 4 in two (7%). Mucositis, nausea and vomiting and diarrhoea occurred in a few patients only. Alterations in liver function tests were also noted, but were usually only alterations in one biochemical parameter. Other minor toxicities (all grade 1 or 2) included somnolence (three cases) and single cases of alopecia, dizziness, dryness of the eyes and general malaise.

### Discussion

The assessment of response to chemotherapy in high-grade gliomas is not easy for a variety of reasons, which include changes of clinical status dependent on steroid dosage, interpretation of imaging results and persistence of clinical disability as a result of the initial tumour and surgical damage. (McDonald *et al.*, 1990). Complete responses are rarely documented because normalisation of the radiological appearance is unlikely as a result of brain destruction due to tumour, surgery and the attendant changes following radiotherapy. The two long-term responders in this study therefore may be considered as worthwhile responses. More difficult to interpret is the significance of the 59% of patients with static disease. Patients who were entered into the study were on a stable steroid dosage and with prior evidence of relapsing tumour. Disease stability may therefore be interpreted as evidence of some anti-tumour activity of the PTX, but this conclusion needs to be accepted with caution.

The most active drugs reported include BCNU, CCNU, MeCCNU, PCNU, procarbazine and dacarbazine, for which

**Table I** Patient characteristics

| | |
|---|---|
| Number of eligible patients entered | 27 |
| Number evaluable[a] | 22 |
| Gender (male/female) | 13/14 |
| Median age (range) | 45 (29–67) |
| WHO performance score 0/1/2/3 | 1/12/8/6 |
| Pathological tumour grade II/III/IV | 1/10/16 |
| *Prior treatment* | |
| Surgery | |
| Biopsy only | 5 |
| Debulking | 22 |
| Radiotherapy | 27 |
| Chemotherapy | 12 |

[a]Five patients are excluded from the response analysis because they received less than 4 weeks' treatment but are included in the toxicity assessment.

**Table II** Toxicity

| *Worst toxicity observed* | *WHO grade* 0 | 1 | 2 | 3 | 4 |
|---|---|---|---|---|---|
| Anaemia | 17 | 7 | 3 | 0 | 0 |
| Leucopenia | 19 | 5 | 3 | 0 | 0 |
| Neutropenia | 22 | 3 | 2 | 0 | 0 |
| Thrombocytopenia | 19 | 1 | 4 | 1 | 2 |
| Mucositis | 23 | 4 | 0 | 0 | 0 |
| Rash | 22 | 5 | 0 | 0 | 0 |
| Nausea/vomiting | 18 | 3 | 5 | 2 | 0 |
| Diarrhoea | 22 | 2 | 1 | 2 | 0 |
| Hepatic[a] | 17 | 3 | 6 | 1 | 0 |

[a]Elevation in one or more of the following parameters: alkaline phosphatase, SGOT, SGPT, or γ-glutamyltransaminase.

response rates of 19–50% have been reported (Lesser *et al.*, 1993). A newer drug, temozolomide, has been reported to give a response in 5/10 (50%) patients (O'Reilly *et al.*, 1993). In this context therefore it may be concluded that PTX has only some limited activity in previously treated high-grade gliomas.

Piritrexim was usually well tolerated, with myelotoxicity being the main side-effect. This was variable and might develop at any time during treatment but usually after dose escalation. This has been previously reported and may indicate variability of absorption (Weiss *et al.*, 1989). Previous chemotherapy, in particular with a nitrosourea, may also have contributed. Thus, of the 12 patients receiving chemotherapy before PTX, toxicities $\geqslant$ grade 2 were seen for total white cell count (WBC) in three (25%) and for platelets in four (33%) patients. In the remaining 15 patients, assessed for toxicity and previously not given chemotherapy, only one grade 2 WBC toxicity was seen (13%). Other toxicities included mucositis, skin rash, mild nausea and vomiting and diarrhoea. All side-effects were rapidly reversible, but treatment was stopped in three patients because of side-effects. The significance of the mild hepatic changes was uncertain. Pulmonary toxicity induced by PTX, as described by De Wit *et al.* (1993*b*), was not seen in this study. There were no drug-related deaths although one patient died in status epilepticus 3 weeks after commencing treatment.

The studies previously reported with PTX in other diseases did not demonstrate better activity than might have been expected from a more conventional antifolate such as methotrexate. Published phase II studies that have reported responses include those with little activity, such as 2/26 (11%) in soft-tissue sarcoma (Schiesel *et al.*, 1992), 5/28 (17%) in head and neck cancer (Vokes *et al.*, 1991), 10/66 (15%) in non-small-cell lung cancer (Kris *et al.*, 1987) and 1/24 (4%) in breast cancer (De Vries *et al.*, 1993). Other studies have reported better response rates in squamous head and neck cancer 9/33 (27%) including three CRs (Uen *et al.*, 1992) and 7/31 (23%) in malignant melanoma with two CRs (Feun *et al.*, 1991). Only in the report on a phase II trial in metastatic urothelial cancer was a high response rate of 11/29 (38%) with one CR seen, but as the authors indicated this is similar to that seen with methotrexate (De Wit *et al.*, 1993*a*). In this present study the expectation that the lipophilicity of PTX might provide an additional drug with a worthwhile response rate in brain tumours, which might then take its place in a combination regimen with a nitrosourea, has not been justified. In spite of the two good responses, the results of this study are inferior to those reported with other drugs currently available.

### References

ADAMSON PC, BALIS FM, MISER J, WELLS RJ, BLEYER WA, WILLIAMS TE, GILLESPIE A, PENTA JS, CLENDENINN NJ AND POPLACK DG. (1990). Pediatric phase I trial and pharmacokinetic study of piritrexim administered orally on a five-day schedule. *Cancer Res.*, **50**, 4464–4467.

DE VRIES EGE, GIETEMA JA, WORKMAN P, SCOTT JE, CRAWSHAW A, DOBBS HJ, DENNIS I, MULDER NH, SLEIJFER D Th AND WILLEMSE PHB. (1993). A phase II pharmacokinetic study with oral piritrexim for metastatic breast cancer. *Br. J. Cancer*, **68**, 641–644.

DE WIT R, KAYE SB, ROBERTS JT, STOTER G, SCOTT J AND VERWEIJ J. (1993*a*). Oral piritrexim, an effective treatment for metastatic urothelial cancer. *Br. J. Cancer*, **67**, 388–390.

DE WIT R, VERWEIJ J, SLINGERLAND R AND STOTER G. (1993*b*). Piritrexim-induced pulmonary toxicity. *Am. J. Clin. Oncol.*, **16**, 146–148.

DUCH DS, EDELSTEIN MP, BOWERS SW AND NICHOLS CA. (1982). Biochemical and chemotherapeutic studies in 2,4-diamino-6 (2,5-dimethoxybenzyl)-5-methylpyrido (2,3d) pyrimidine (BW30lu). A novel lipid-soluble inhibitor of dihydrofolate reductase. *Cancer Res.*, **42**, 3987–3994.

FEUN LG, GONZALEZ R, SAVARAJ N, HANLON J, COLLIER M, ROBINSON WA AND CLENDENINN NJ. (1991). Phase II trial of piritrexim in metastatic melanoma using intermittent, low-dose administration. *J. Clin. Oncol.*, **9**, 464–467.

KRIS MG, GRALLA RJ, BURKE T, BERKOWITZ LD, MARKS LD, KELSEN DP AND HEELAN RT. (1987). Phase II trial of oral piritrexim (BW301U) in patients with stage III non-small cell lung cancer. *Cancer Treat. Rep.*, **71**, 763–764.

LESSER J AND GROSSMAN SA. (1993). The chemotherapy of adult primary brain tumors. *Cancer Treat. Rev.*, **19**, 261–281.

MACDONALD DR, CASCINO TL, SCHOLD JR SC AND CAIRNCROSS JG. (1990). Response criteria for phase II studies of supratentorial malignant glioma. *J. Clin. Oncol.*, **8**, 1277–1280.

MARSHALL M, VON HOFF D, CHACKO A AND WILLIAMS T. (1985). Effects of drug concentration, exposure time, and serum dialysis on antitumour activity of BW301U in the human tumour closing assay (abstract). *Proc. Am. Assoc. Cancer Res.*, **26**, 364.

NEUENFELDT B, VON HOFF D, WHITECAR J AND WILLIAMS T. (1982). Comparison of activity of lipid-soluble pyrido-pyrimidine BW301U and methotrexate (MTX) against human colony forming units (TCFUs). *Proc. Am. Assoc. Cancer Res.*, **23**, 181.

O'REILLY SM, NEWLANDS ES, GLASER MG, BRAMPTON M, RICE-EDWARDS JM, ILLINGWORTH RD, RICHARDS PG, KENNARD C, COLQUHOUN IR, LEWIS P AND STEVENS MFG. (1993). Temozolomide: a new oral cytotoxic chemotherapeutic agent with promising activity against primary brain tumours. *Eur. J. Cancer*, **29A**, 940–942.

SCHIESEL, JD, CARABASI M, MAGILL G, CASPER E, CHENG E, MARKS L, FEYZI J, CLENDENINN NJ AND SMALLEY RV. (1992). Oral piritrexim – a phase II study in patients with advanced soft tissue sarcoma. *Invest. New Drugs*, **10**, 97–98.

SEDWICK WD, HAMRELL M, BROWN OE AND LAZLO J. (1982). Metabolic inhibition by a new antifolate 2,4-diamino-6 (2,5-dimethoxybenzyl)-5-methylpyrido (2,3d) pyrimidine (BW30lu), an effective inhibitor of human lymphoid and dehydrofolate reductase-overproducing mouse cell lines. *Mol. Pharmacol.*, **22**, 766–770.

SIGEL CW, MACKLIN AW, WOOLLEY JL, JOHNSON NW, COLLIER MA, BLUM MR, CLENDENINN NJ, EVERITT BJM, GREBE G, MACKARS A, FOSS RG, DUCH DS, BOWERS SW AND NICHOL CA. (1987). Preclinical biochemical pharmacology and toxicology of piritrexim, a lipophile inhibitor of dehydrofolate reductase. *NCI Monogr.* **5**, 111–120.

UEN W-C, HUANG AT, MENNEL R, JONES SE, SPAULDING MB, KILLION K, HAVLIN K, KEEGAN P AND CLENDENINN NJ. (1992). A phase II study of piritrexim in patients with advanced squamous head and neck cancer. *Cancer*, **69**, 1088–2011.

VOKES EE, DIMERY IW, JACOBS CD, KARP D, MOLINA A, COLLIER MA, EBLE ML AND CLENDENINN NJ. (1991). A phase II study of piritrexim in combination with methotrexate in recurrent and metastatic head and neck cancer. *Cancer*, **67**, 2253–2257.

WEISS GR, SAROSY AG, SHENKENBERG TD, WILLIAMS T, CLENDENINN NJ, VON HOFF DD, WOOLLEY JL, LIAO SHT AND BLUM MR. (1989). A phase I clinical and pharmacological study of weekly intravenous infusions of piritrexim (BW301U). *Eur. J. Cancer Clin. Oncol.*, **25**, 1867–1873.

WORLD HEALTH ORGANIZATION. (1979). *WHO Handbook for Reporting Results of Cancer Treatment*. WHO: Geneva.


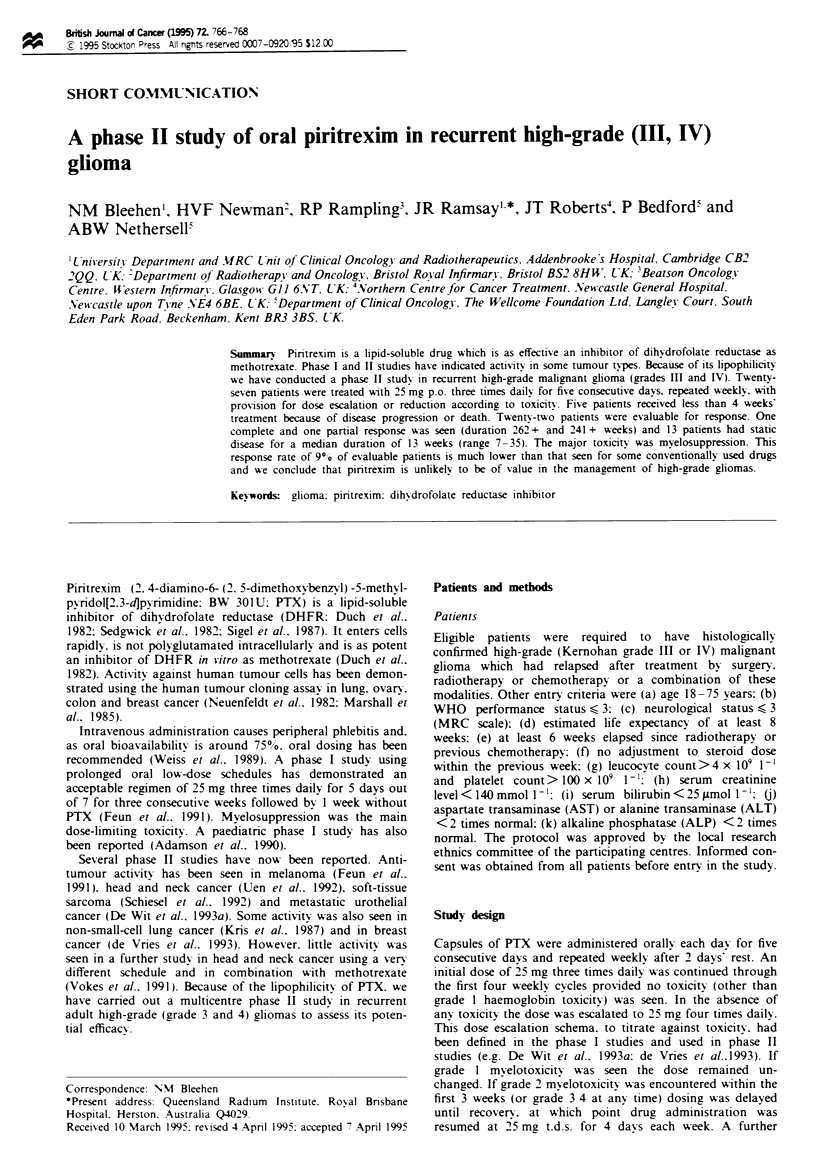

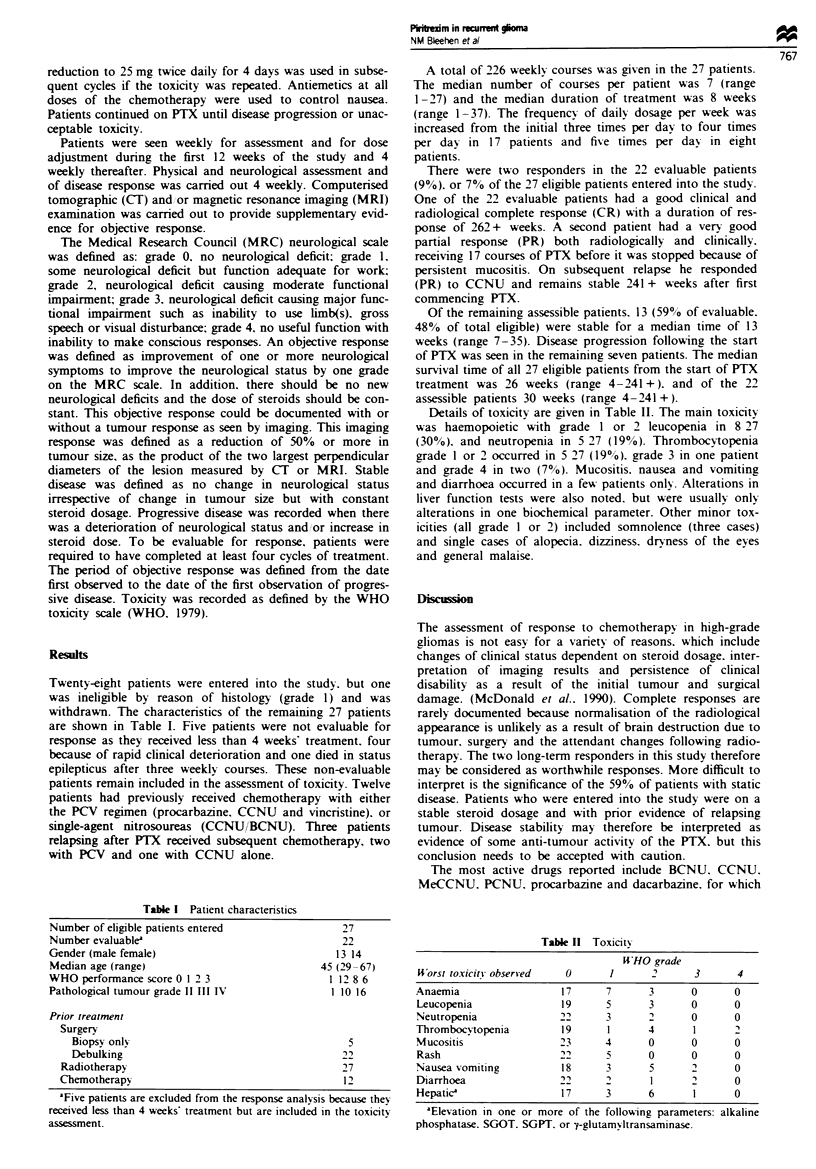

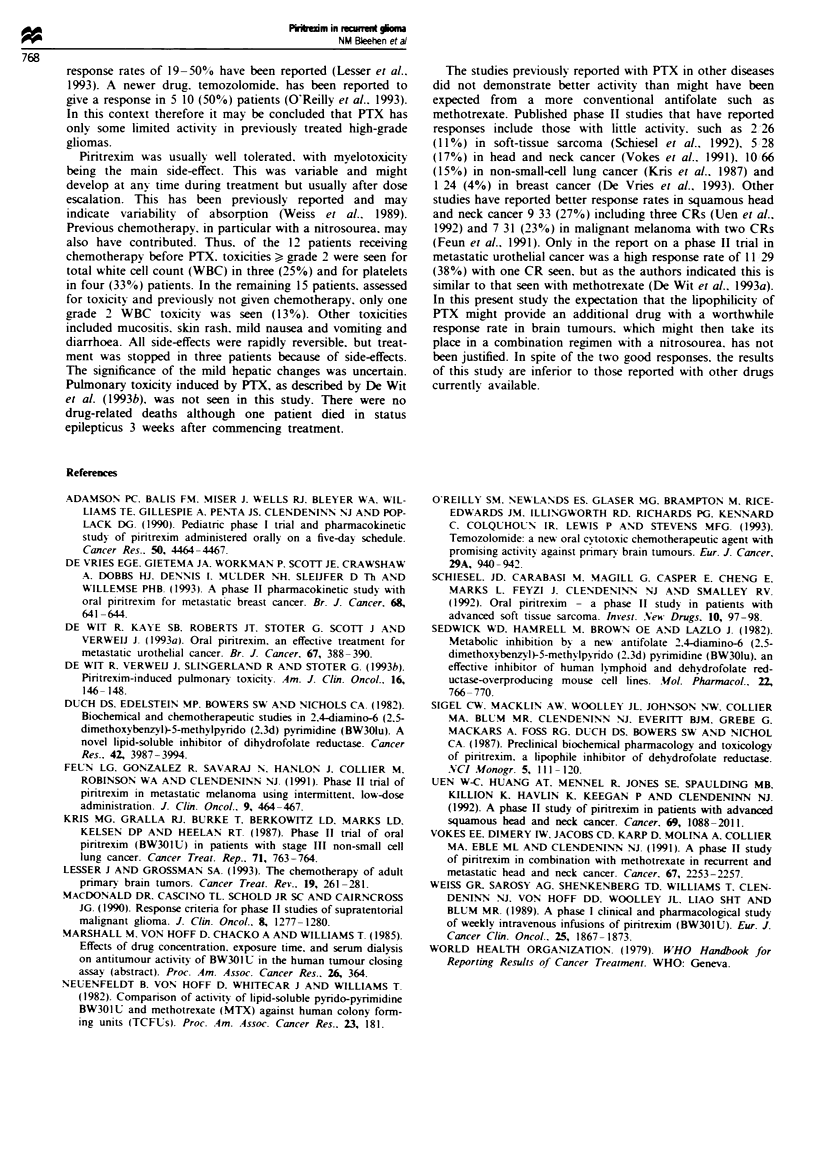


## References

[OCR_00340] Adamson P. C., Balis F. M., Miser J., Wells R. J., Bleyer W. A., Williams T. E., Gillespie A., Penta J. S., Clendeninn N. J., Poplack D. G. (1990). Pediatric phase I trial and pharmacokinetic study of piritrexim administered orally on a five-day schedule.. Cancer Res.

[OCR_00366] Duch D. S., Edelstein M. P., Bowers S. W., Nichol C. A. (1982). Biochemical and chemotherapeutic studies on 2,4-diamino-6-(2,5-dimethoxybenzyl)-5-methylpyrido[2,3-d]pyrimidine (BW 301U), a novel lipid-soluble inhibitor of dihydrofolate reductase.. Cancer Res.

[OCR_00371] Feun L. G., Gonzalez R., Savaraj N., Hanlon J., Collier M., Robinson W. A., Clendeninn N. J. (1991). Phase II trial of piritrexim in metastatic melanoma using intermittent, low-dose administration.. J Clin Oncol.

[OCR_00380] Kris M. G., Gralla R. J., Burke M. T., Berkowitz L. D., Marks L. D., Kelsen D. P., Heelan R. T. (1987). Phase II trial of oral piritrexim (BW301U) in patients with stage III non-small cell lung cancer.. Cancer Treat Rep.

[OCR_00385] Lesser G. J., Grossman S. A. (1993). The chemotherapy of adult primary brain tumors.. Cancer Treat Rev.

[OCR_00389] Macdonald D. R., Cascino T. L., Schold S. C., Cairncross J. G. (1990). Response criteria for phase II studies of supratentorial malignant glioma.. J Clin Oncol.

[OCR_00404] O'Reilly S. M., Newlands E. S., Glaser M. G., Brampton M., Rice-Edwards J. M., Illingworth R. D., Richards P. G., Kennard C., Colquhoun I. R., Lewis P. (1993). Temozolomide: a new oral cytotoxic chemotherapeutic agent with promising activity against primary brain tumours.. Eur J Cancer.

[OCR_00412] Schiesel J. D., Carabasi M., Magill G., Casper E., Cheng E., Marks L., Feyzi J., Clendeninn N. J., Smalley R. V. (1992). Oral piritrexim--a phase II study in patients with advanced soft tissue sarcoma.. Invest New Drugs.

[OCR_00418] Sedwick W. D., Hamrell M., Brown O. E., Laszlo J. (1982). Metabolic inhibition by a new antifolate, 2,4-diamino-6-(2,5-dimethoxybenzyl)-5-methyl-pyrido[2,3-d]pyrimidine (BW3O1U), an effective inhibitor of human lymphoid and dihydrofolate reductase-overproducing mouse cell lines.. Mol Pharmacol.

[OCR_00426] Sigel C. W., Macklin A. W., Woolley J. L., Johnson N. W., Collier M. A., Blum M. R., Clendeninn N. J., Everitt B. J., Grebe G., Mackars A. (1987). Preclinical biochemical pharmacology and toxicology of piritrexim, a lipophilic inhibitor of dihydrofolate reductase.. NCI Monogr.

[OCR_00437] Vokes E. E., Dimery I. W., Jacobs C. D., Karp D., Molina A., Collier M. A., Eble M. L., Clendeninn N. J. (1991). A phase II study of piritrexim in combination with methotrexate in recurrent and metastatic head and neck cancer.. Cancer.

[OCR_00446] Weiss G. R., Sarosy G. A., Shenkenberg T. D., Williams T., Clendeninn N. J., Von Hoff D. D., Woolley J. L., Liao S. H., Blum M. R. (1989). A phase I clinical and pharmacological study of weekly intravenous infusions of piritrexim (BW301U).. Eur J Cancer Clin Oncol.

[OCR_00347] de Vries E. G., Gietema J. A., Workman P., Scott J. E., Crawshaw A., Dobbs H. J., Dennis I., Mulder N. H., Sleijfer D. T., Willemse P. H. (1993). A phase II and pharmacokinetic study with oral piritrexim for metastatic breast cancer.. Br J Cancer.

[OCR_00356] de Wit R., Kaye S. B., Roberts J. T., Stoter G., Scott J., Verweij J. (1993). Oral piritrexim, an effective treatment for metastatic urothelial cancer.. Br J Cancer.

[OCR_00361] de Wit R., Verweij J., Slingerland R., Stoter G. (1993). Piritrexim-induced pulmonary toxicity.. Am J Clin Oncol.

